# Supplementary material for: Exploration of cell state heterogeneity using single-cell proteomics through sensitivity-tailored data-independent acquisition
Source: Nat Commun. 2023 Sep 22;14:5910. doi: 10.1038/s41467-023-41602-1 (PMC10517177; doi:10.1038/s41467-023-41602-1)
Supplement: Supplementary file 1 — Supplementary Information [file 41467_2023_41602_MOESM1_ESM.pdf]

Supplementary Information

Exploration of cell state heterogeneity using single-cell proteomics through sensitivity-tailored data-independent acquisition

Petrosius et al.

A

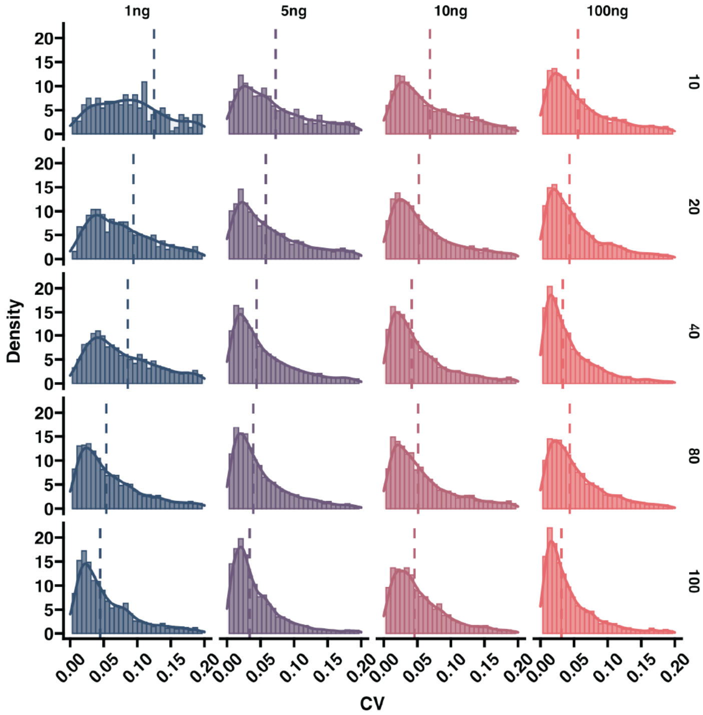

Supplementary Figure 1. Higher resolution/IT improves quantification precision. A) Coefficient of variation (CV) distribution histograms with different peptides load and DIA isolation windows. Related to Figure 1. Source data are provided as a Source Data file.

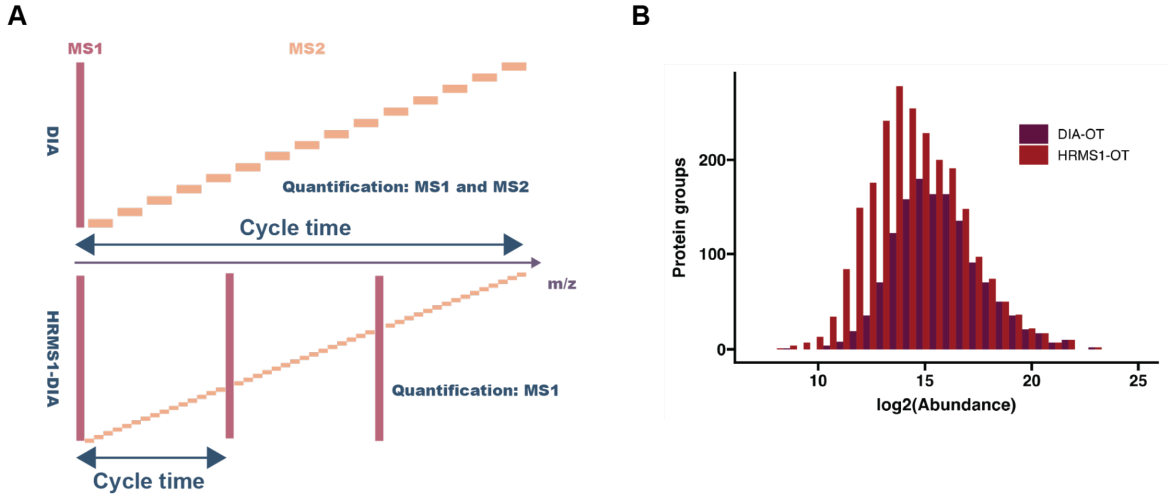

C

DIA

Resolution		Window		
MS1	MS2	Size	Number	Cycle-time
120K	15K	15mz	40	1.536s
120K	30K	30mz	20	1.536s
120K	60K	60mz	10	1.536s
240K	120K	120mz	5	1.536s

HRMS1

Resolution		Window		
MS1	MS2	Size	Number	Cycle-time
120K	30K	10mz	60	1.536s
120K	60K	20mz	30	1.536s
120K	120K	40mz	15	1.536s
240K	240K	100mz	6	1.280s

Supplementary Figure 2. Supporting figures and tables for HRMS1. A) Schematic illustration of standart DIA and HRMS1 acquisition. B) Histogram showing detected number of proteins at a certain log transformed abundance bin with HRMS1 or standard DIA. C) Tables summarizing the resolution and isolation window parameters used for the window survey. Related to Figure 2. Source data are provided as a Source Data file.

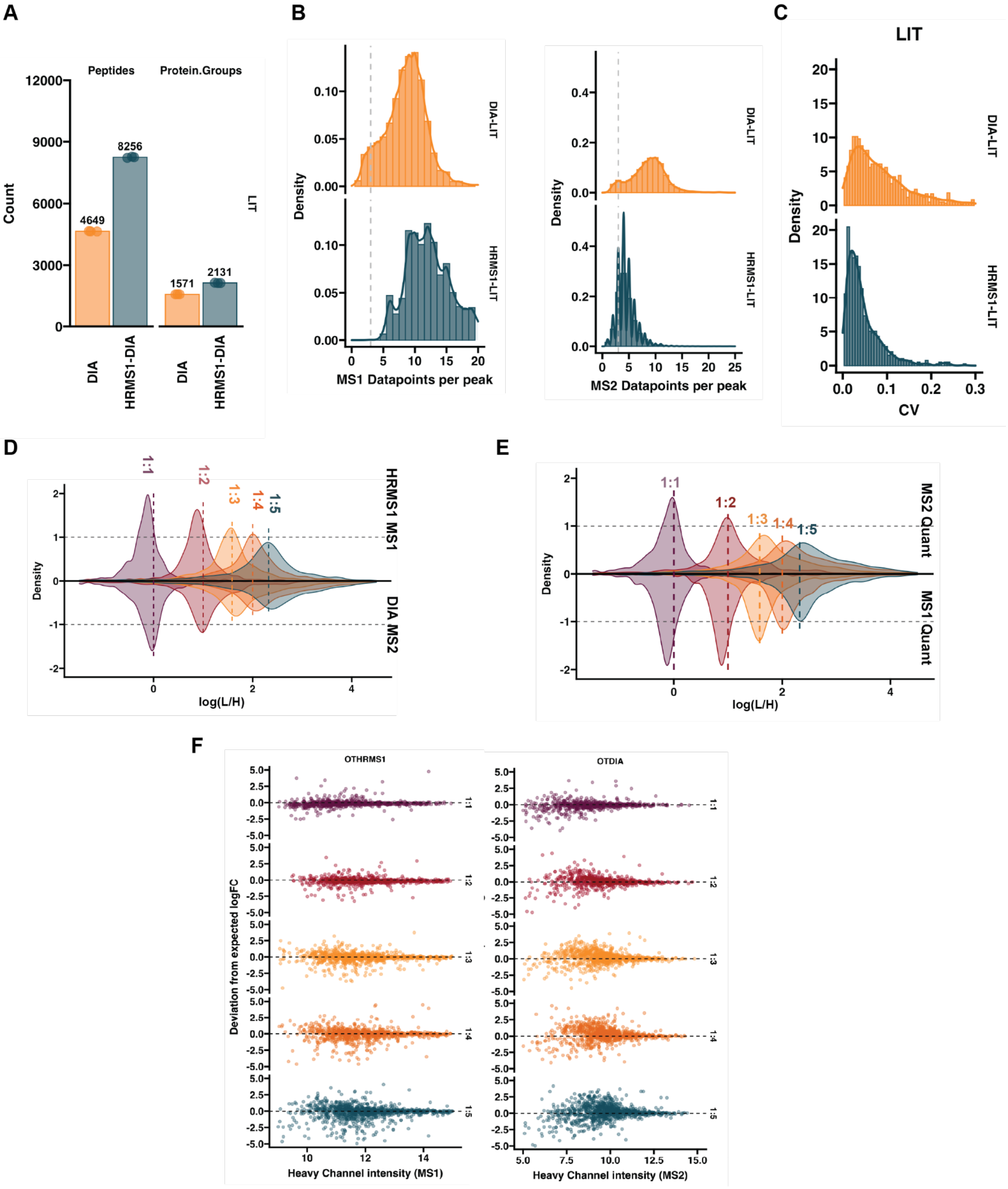
A
B
C
D
E
F
Peptides
Protein.Groups
Count
LIT
4649
8256
1571
2131
DIA
HRMS1-DIA
Density
DIA-LIT
HRMS1-LIT
MS1 Datapoints per peak
MS2 Datapoints per peak
CV
HRMS1 MS1
DIA MS2
log(L/H)
MS2 Quant
MS1 Quant
1:1
1:2
1:3
1:4
1:5
OTHRMS1
OTDIA
Deviation from expected logFC
Heavy Channel intensity (MS1)
Heavy Channel intensity (MS2)

Supplementary Figure 3. Wide isolation window application to LIT-DIA and MS1 quantification accuracy. A) Barplot showing detected number of proteins and peptides with LIT-DIA or HRMS1-LIT-DIA B) Histogram showing data points per peak on MS1 (left) and MS2 (right) level with both methods. C) Histogram of CV values for protein quantification. D-E) Density plots showing different SILAC light and heavy mixes. In D) the ratios between MS1 level quant in HRMS1-DIA and MS2 level in standart DIA are compared. In E) MS2 (top) and MS1 (bottom) quantification is directly compared from the same standart DIA method. Dashed lines indicated expected peptide mix values. Related to Figure 2. F) Scatter plot where the measurement error is plotted on the y-axis and the log2 transformed heavy protein abundance on the x-axis. Left side represent HRMS1-DIA and right standart DIA quantification. Source data are provided as a Source Data file.

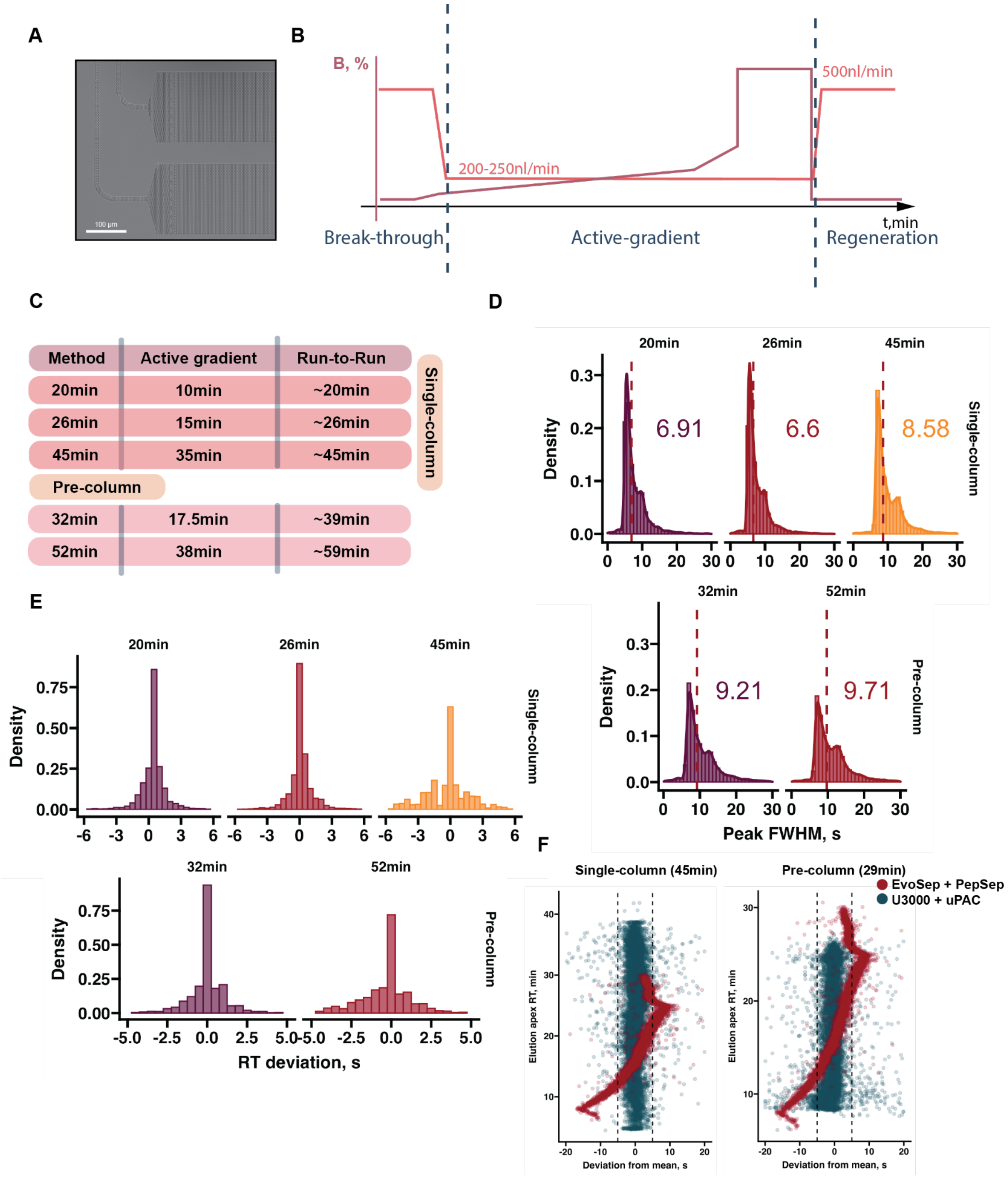
A
100 µm
B
B, %
500nl/min
200-250nl/min
t,min
Break-through
Active-gradient
Regeneration
C
Method | Active gradient | Run-to-Run
20min | 10min | ~20min
26min | 15min | ~26min
45min | 35min | ~45min
Single-column
Pre-column
32min | 17.5min | ~39min
52min | 38min | ~59min
D
20min
26min
45min
Density
6.91
6.6
8.58
Single-column
32min
52min
9.21
9.71
Pre-column
Peak FWHM, s
E
20min
26min
45min
Density
Single-column
32min
52min
Pre-column
RT deviation, s
F
Single-column (45min)
Pre-column (29min)
EvoSep + PepSep
U3000 + uPAC
Elution apex RT, min
Deviation from mean, s

Supplementary Figure 4. Chromatographic performance of the uPAC Neo limited-sample analytical column. A) Scanning electron microscopy (SEM) image of the micropillar-column. B) Schematic visualizing the gradient used with the uPAC column, including flow-ramping capability of the Ultimate3000. C) Table summarizing the used methods. D) Histograms of peak FWHM with different method lengths with the single-column or pre-column configuration. E) Histogram showing the peptide elution peak apex deviation from a mean calculated from three replicates. The deviations of one replicate from the mean are shown. The plotted peak parameters were obtained from 5ng DIA runs analyzed with Spectronaut16. Related to Figure 3. F) Comparison of retention time (RT) stability with EvoSep-PepSep and U300-uPAC LC set-ups. The uPAC single-column or precolumn configuration is noted above the plots.The deviation of a single replicate (n=2) from the mean RT time of 3 replicas is plotted on the x-axis and the peptide elution peak apex time on the y axis. The different LC setups are indicated by color. Data for 1ng injection used in both comparisons. Source data are provided as a Source Data file.

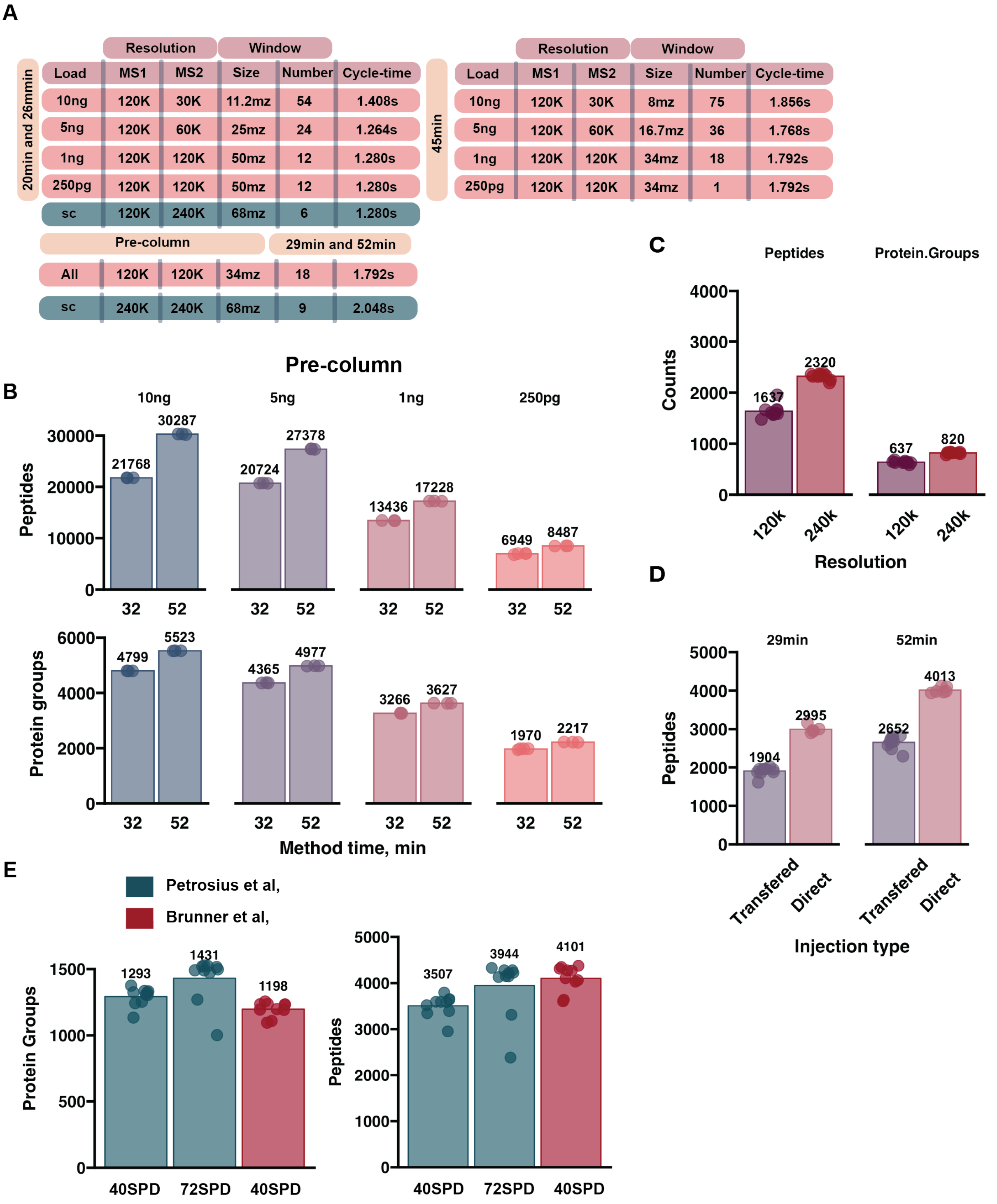

Supplementary Figure 5. uPAC column benchmarking supporting information. A) Table summarise all the acquisition method used with the uPAC Neo Low Load analytical column. Methods colored in blue note the ones used for single-cell (sc) input. The complete optimization data can be found in Table S2. B) Barplot showing detected number of proteins and peptides with the pre-column configuration C) Barplot showing the number of quantified peptides and protein groups with the 120k and 240k WISH-DIA methods. D) Barplot showing the number of quantified peptides from single-cell input with pre-column configuration, direct injection from 384 well plate (direct) or transferred to 96 well (Transferred)

E) Bar of detected number of proteins (left) and peptides (right) from our study and Brunner et al, 2022. Related to Figure 3. Source data are provided as a Source Data file.

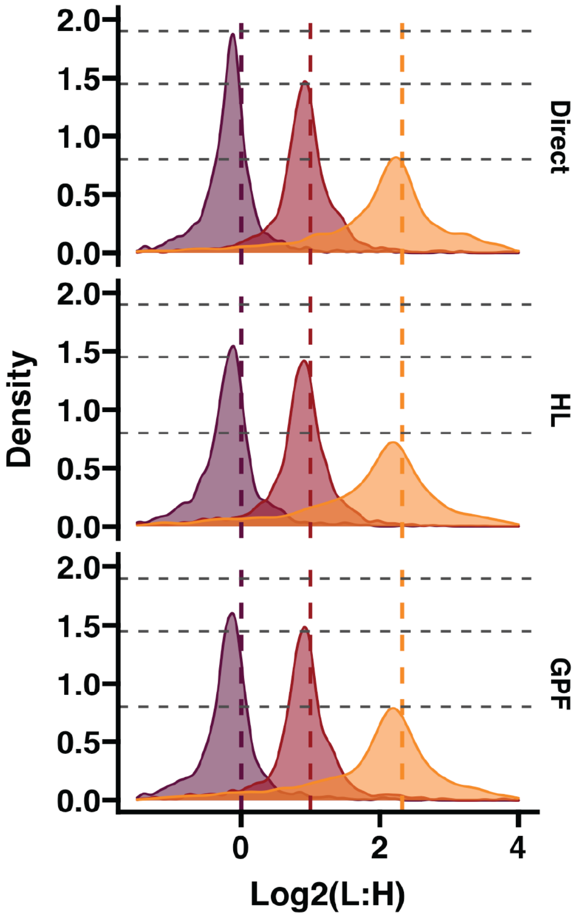

Supplementary Figure 6. DirectDIA detected protein accuracy with and without a library. Density plots showing the log2 transformed light and heavy protein abundance ratios. Proteins quantified with directDIA shown in top and high-load (HL) library in the middle and gas-phase fractionated (GPF) in the bottom. Dashed lines denote expected ratios. MS1 based quantification used. Related to Figure 4. Source data are provided as a Source Data file.

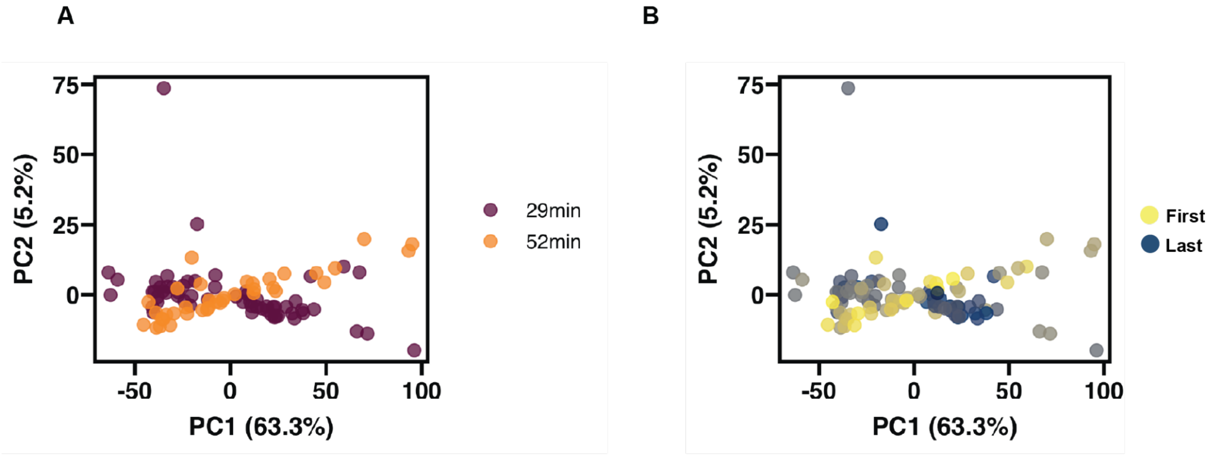

Supplementary Figure 7. Principal component analysis of single-cell datasets. Clustering of the integrated single-cell with PCA. Principal components 1 and 2 are shown. A) Color coding marks the gradient used for the single-cell runs. B) Marks the order in which the samples were run. 52min runs were carried out after 29min. Related to Figure 5. Source data are provided as a Source Data file.

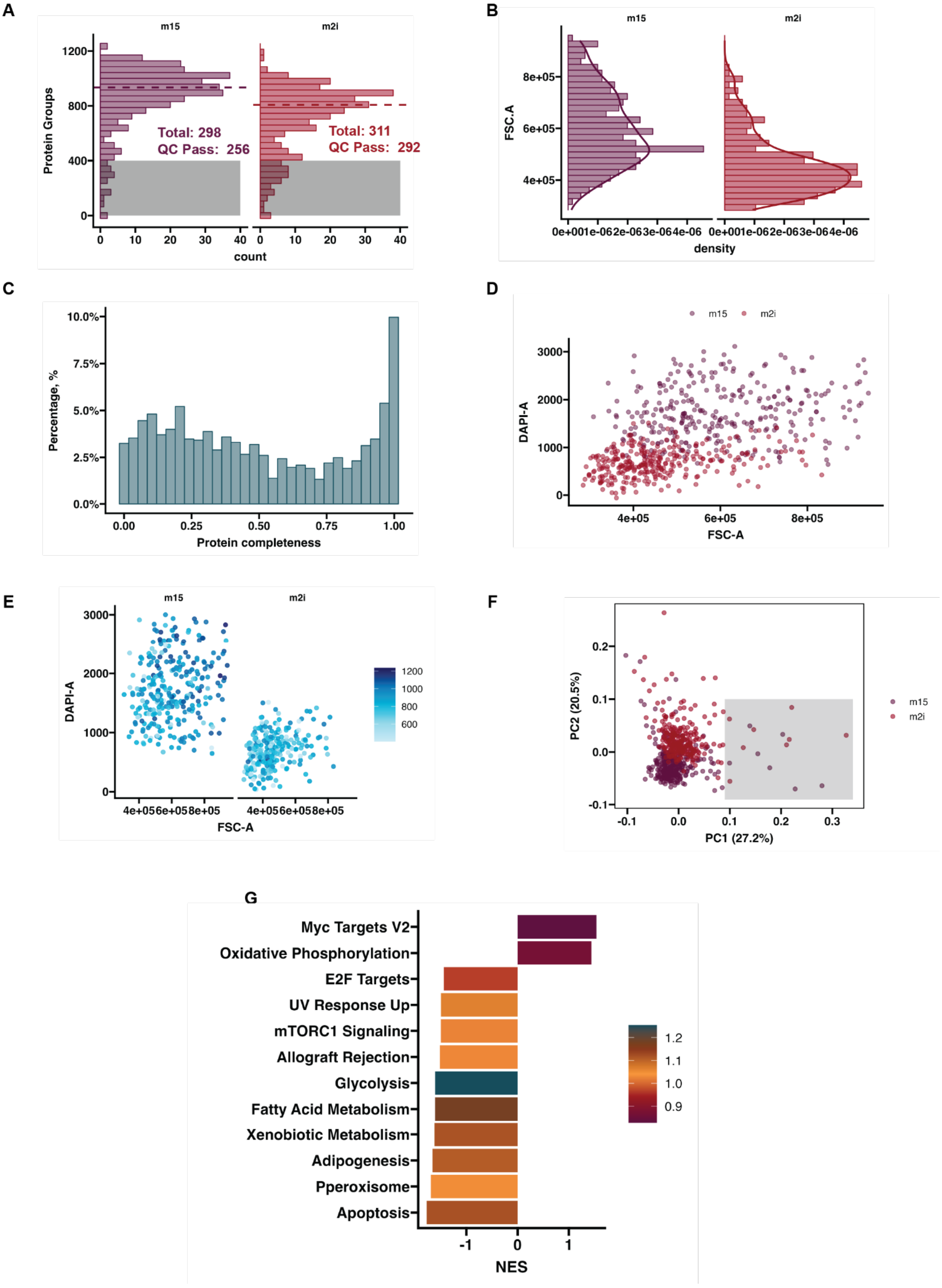

A
m15
m2i
Protein Groups
count
Total: 298
QC Pass: 256
Total: 311
QC Pass: 292
B
m15
m2i
FSC.A
density
C
Percentage, %
Protein completeness
D
m15
m2i
DAPI-A
FSC-A
E
m15
m2i
DAPI-A
FSC-A
F
PC2 (20.5%)
PC1 (27.2%)
m15
m2i
G
Myc Targets V2
Oxidative Phosphorylation
E2F Targets
UV Response Up
mTORC1 Signaling
Allograft Rejection
Glycolysis
Fatty Acid Metabolism
Xenobiotic Metabolism
Adipogenesis
Pperoxisome
Apoptosis
NES

Supplementary Figure 8. Quality control figures for mESC A) Histogram of proteome coverage for m2i and m15 cells.Grey block indicates the cell region that was removed from further analysis. B) Histogram showing cell forward-side scatter are (FSC-A) distribution. C) Protein wise completeness of the generated data. D) Scatter plot showing the size and DAPI-Area correlation. E) Scatter plot showing DAPI-Area and FSC-A correlation with color code indicating detected number of protein groups. F) PCA of cells that passed the initial QC filter. Cell in the gray block are removed from further analysis. Related to Figure 6 and 7. Source data are provided as a Source Data file.

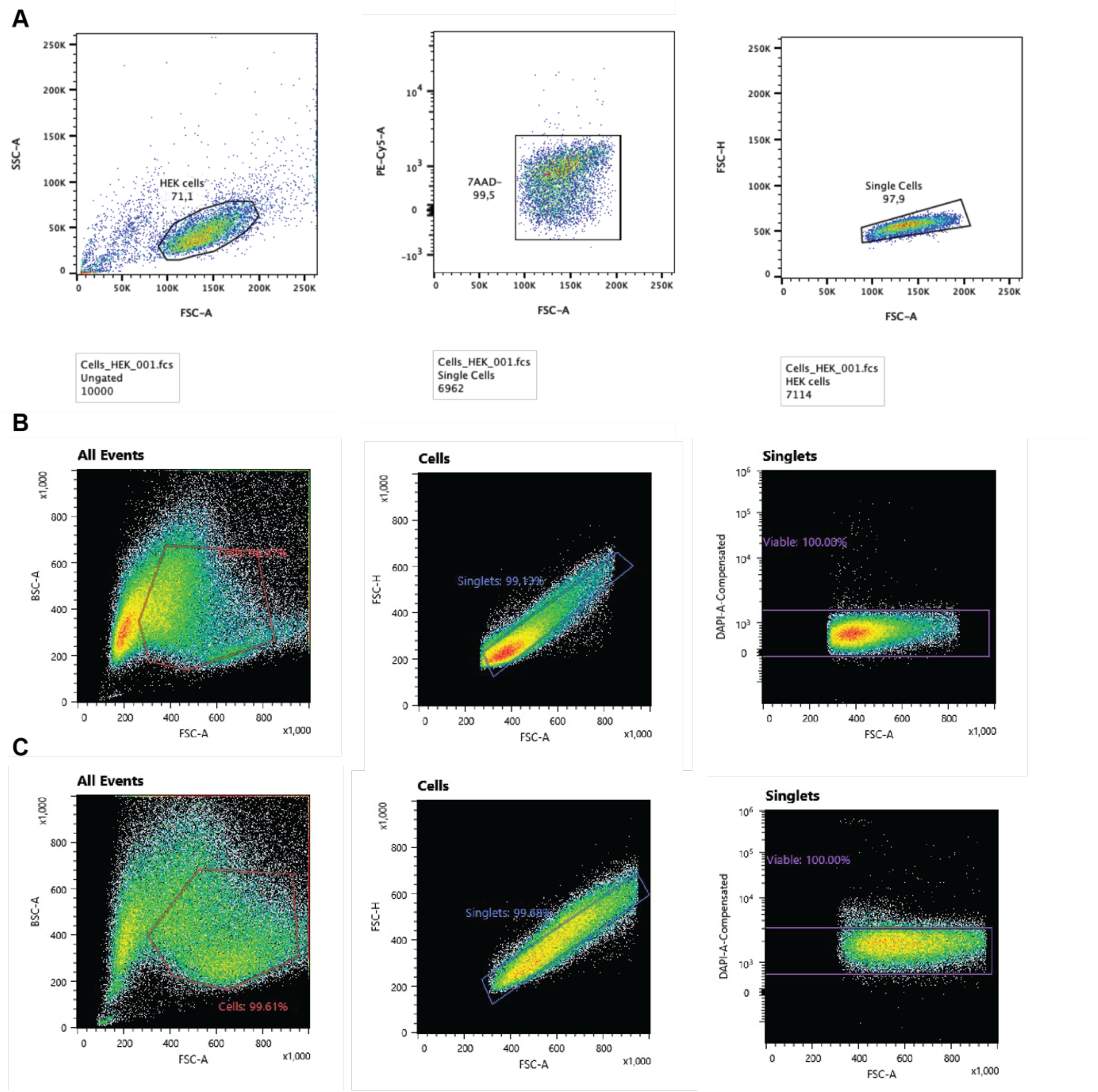

Supplementary Figure 9. FACS gating schemes for single-cell sample preparation A) HEK293 B) mESC m2i and C) mESC m15. SSC-A – side scatter area, FSC-A forward scatter area and BSC-A back scatter area. PE-Cy5A and DAPI are used to select for live cells.
