# Supplementary material for: Exploration of cell state heterogeneity using single-cell proteomics through sensitivity-tailored data-independent acquisition
Source: Nat Commun. 2023 Sep 22;14:5910. doi: 10.1038/s41467-023-41602-1 (PMC10517177; doi:10.1038/s41467-023-41602-1)
Supplement: Supplementary file 3 — Description of Additional Supplementary files [file 41467_2023_41602_MOESM3_ESM.docx]

File Name: Supplementary Data 1

Description: Contains summarized Spectronaut output for the DIA window optimization assay with the EvoSep One - PepSep scheme. Related to Figure 1

File Name: Supplementary Data 2

Description: Contains summarized Spectronaut output for the DIA window optimization assay with the U3000-uPAC scheme. Related to Figure 3
